# Reevaluating PRRSV-induced respiratory distress: beyond interstitial pneumonia to pulmonary surfactant dysfunction and airway obstruction

**DOI:** 10.3389/fimmu.2026.1720057

**Published:** 2026-03-19

**Authors:** Hongbo Chen, Xiaobing Li, Chong Cao, Longxin Qiu, Dianning Duan

**Affiliations:** 1 College of Life Sciences, Longyan University, Longyan, Fujian, China; 2 Fujian Engineering Research Center for Swine Disease Control and Prevention, Longyan, Fujian, China

**Keywords:** alveolar collapse, mucus hypersecretion, porcine reproductive and respiratory syndrome virus, pulmonary surfactant protein, respiratory distress, ventilatory dysfunction

## Introduction

Porcine Reproductive and Respiratory Syndrome Virus (PRRSV), a member of the Arteriviridae family, continues to challenge global swine production through its genetic variability and complex pathogenesis (1–6). The conventional understanding attributes PRRSV-induced respiratory symptoms primarily to viral targeting of alveolar macrophages, which triggers severe interstitial pneumonia and inflammatory responses that compromise gas exchange across the alveolar-capillary interface (7). Additionally, research indicates that PRRSV activates oxidative stress through the TLR4/NF-κB pathway, further contributing to tissue damage (8). This perspective has led to therapeutic strategies predominantly focused on inflammation control.

Alveolar macrophages, as the primary host cells for PRRSV, experience numerical reduction and functional impairment post-infection, leading to immunosuppression and significant inflammation (9, 10). Nevertheless, the classical theory cannot fully explain clinical observations: why does respiratory distress persist after inflammation is controlled? Why does the severity of respiratory distress sometimes not correlate with the degree of pulmonary inflammation in certain cases? This study proposes that PRRSV’s targeted disruption of the pulmonary surfactant system is a key driver of respiratory distress, representing a paradigm shift from “inflammation-centric” to “ventilatory mechanics failure-centric“ models. Below, we elaborate the scientific foundation of this new perspective and its potential transformative significance for treating PRRSV respiratory symptoms.

## Inadequacies of the traditional interstitial pneumonia theory

Classical pathology primarily attributes PRRSV-induced respiratory distress to interstitial pneumonia and secondary infections resulting from immunosuppression. According to this view, PRRSV infection of alveolar macrophages triggers massive release of pro-inflammatory cytokines, recruiting inflammatory cell infiltration, thickening alveolar septa, increasing gas exchange distance, and ultimately leading to dyspnea (11, 12). Indeed, studies confirm that PRRSV infection causes characteristic interstitial pneumonia, manifested as alveolar septal thickening, mononuclear cell infiltration, and type II epithelial cell hyperplasia (13, 14). However, several clinical and experimental observations challenge this traditional view. First, in some PRRSV cases, the severity of respiratory distress does not fully correlate with the degree of pulmonary inflammation, suggesting factors beyond inflammation drive dyspnea. Second, early-onset respiratory distress can be a prominent initial symptom of PRRSV infection, preceding severe gas exchange impairment caused by extensive interstitial pneumonia. Third, there is frequently an inadequate treatment response: controlling inflammation and secondary infections often fails to rapidly alleviate respiratory distress, indicating other unaddressed pathological mechanisms.

## Core elements of the new paradigm: pulmonary surfactant system failure

Emerging research indicates that PRRSV’s destructive impact on lung tissue extends beyond traditional understanding. Studies demonstrate that PRRSV infection dysregulates tight junction protein expression, promoting increased vascular permeability and acute lung injury (11). Crucially, PRRSV directly or indirectly damages alveolar epithelial type II cells (AECIIs)—the primary cells responsible for synthesizing and secreting pulmonary surfactant, particularly surfactant proteins B and C (SP-B, SP-C) (15, 16), through two complementary pathways (1): low-level direct infection (requiring confirmation via more precise *in vivo* evidence); and (2) more importantly, indirect ‘bystander’ injury mediated by inflammatory cytokines (e.g., IL-1β, TNF-α) released from infected alveolar macrophages and the ensuing oxidative stress microenvironment, leading to AECII dysfunction and apoptosis. Pulmonary surfactant is essential for reducing alveolar surface tension and preventing end-expiratory alveolar collapse. By suppressing SP-B and SP-C expression, PRRSV causes a sharp increase in alveolar surface tension, predisposing alveoli to collapse (atelectasis). This forces the respiratory system to expend greater effort to re-expand collapsed alveoli, clinically manifesting as severe dyspnea and tachypnea—a “compliance reduction” type respiratory failure. Simultaneously, PRRSV infection may stimulate specific inflammatory pathways, promoting goblet cell hyperplasia and hypersecretion, forming viscous mucus plugs that physically obstruct small airways (17, 18). Oxidative stress further amplifies this damage by directly attacking cell membrane lipids, proteins, and DNA, leading to dysfunction and death of AECIIs and endothelial cells, thereby exacerbating both surfactant synthesis impairment and endothelial barrier damage (19, 20). Oxidative stress acts as a critical “injury amplifier,” closely linking PRRSV’s direct effects with subsequent inflammatory responses.

**Figure 1:**
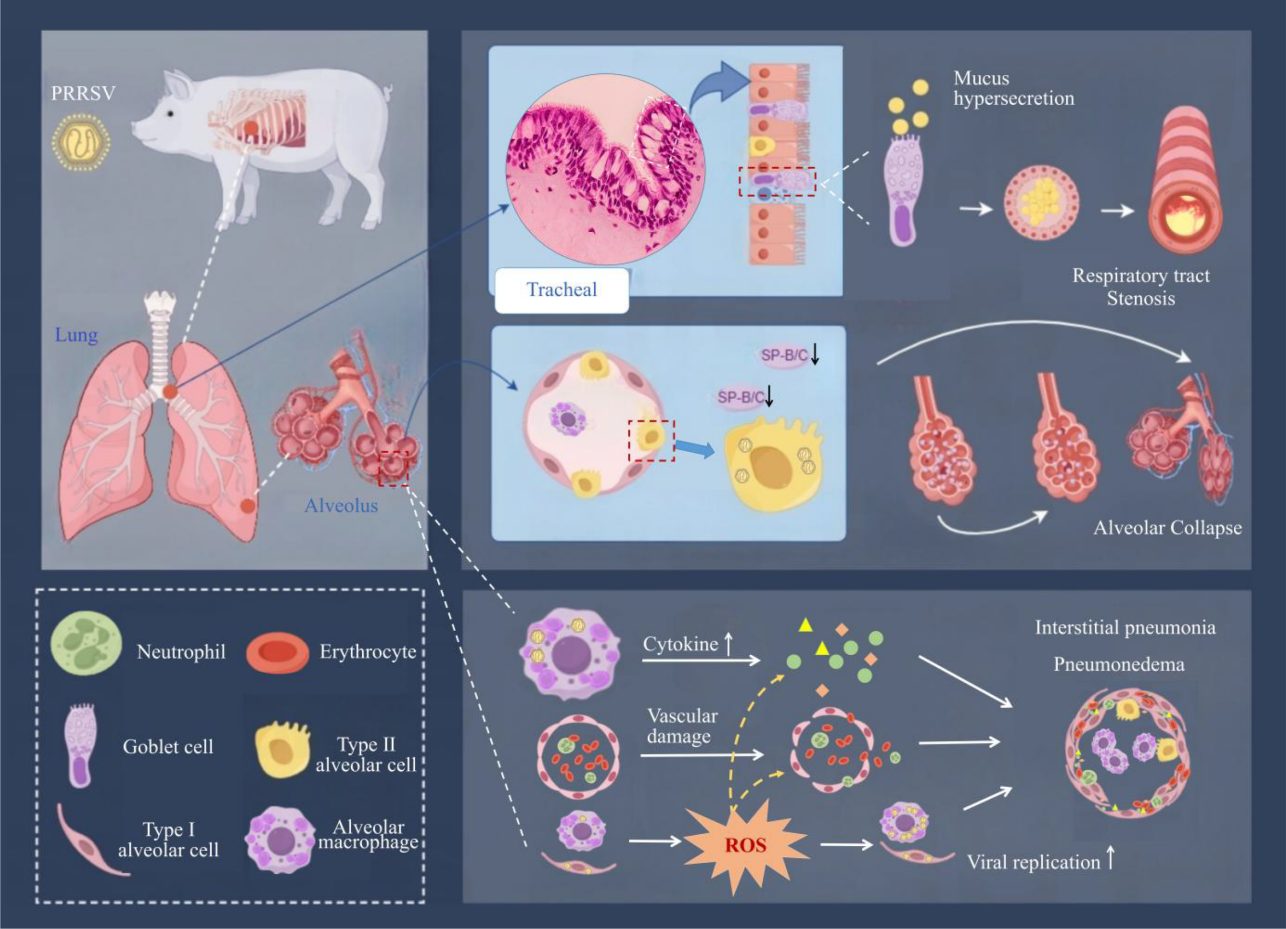
Multiple mechanisms and interrelationships of PRRSV induced respiratory distress. This figure was created using Figdraw.

We propose that an earlier, more central driver of respiratory distress following PRRSV infection is “ventilatory mechanics” failure, mediated by two synergistic mechanisms (1). Pulmonary Surfactant System Collapse: PRRSV directly or indirectly impairs AECIIs function, inhibiting the synthesis and secretion of pulmonary surfactant (particularly SP-B, SP-C), leading to sharply increased alveolar surface tension and end-expiratory alveolar collapse (2). Airway Mucus Hypersecretion and Physical Obstruction: PRRSV infection activates specific inflammatory pathways, stimulating airway goblet cell hyperplasia and hypersecretion, forming viscous mucus plugs that physically obstruct small airways. These two mechanisms collectively cause “mixed ventilatory mechanics failure”—concurrent “failure to open” alveoli (restrictive ventilatory defect) and “blocked” small airways (obstructive ventilatory defect). This mechanism can emerge early in the disease course, potentially well before severe gas exchange impairment from interstitial pneumonia (**Figure 1**).

## PRRSV disruption of the pulmonary surfactant system

Pulmonary surfactant is a complex mixture synthesized and secreted by AECIIs, wherein surfactant proteins (SP-A, SP-D, SP-B, SP-C) and phospholipids collectively maintain alveolar stability (21, 22). Notably, SP-A and SP-D are not only physical factors reducing surface tension but also crucial innate immune molecules playing key roles in pulmonary immune defense. We propose PRRSV disrupts this system through two synergistic mechanisms. 1 Potential Direct Mechanism: Although definitive *in vivo* evidence is still needed, PRRSV may directly infect a subset of AECIIs, potentially inhibiting surfactant protein synthesis and secretion. Studies show disordered surfactant protein expression in lung tissues of PRRSV-infected pigs. Compared to controls, SP-A expression increases non-significantly, SP-D expression significantly increases, while SP-C expression significantly decreases in PRRSV-infected porcine lungs. This dysregulation disrupts normal alveolar surfactant system function (22). 2 Indirect Mechanism (predominant): PRRSV infection induces immunometabolic reprogramming and releases pro-inflammatory cytokines (e.g., IL-1β, TNF-α) from infected macrophages, creating a hostile microenvironment that impairs AECII metabolism and deprives these cells of energy and substrates required for surfactant synthesis. Research indicates PRRSV infection upregulates microRNA ssc-miR-185 targeting claudin-8 to inhibit its post-transcriptional expression; similar mechanisms may regulate the surfactant protein system (11).

The primary function of pulmonary surfactant is to reduce surface tension at the alveolar air-liquid interface, preventing end-expiratory alveolar collapse (23). When PRRSV inhibits surfactant production, it induces a series of alveolar ventilatory mechanics failure. SP-B and SP-C, as hydrophobic proteins, maintain lipid distribution on the alveolar surface and reduce surface tension. Their downregulation directly increases alveolar surface tension. Increased surface tension predisposes alveoli to collapse during expiration, significantly reducing lung compliance. The body requires greater effort to re-expand collapsed alveoli, clinically presenting as severe dyspnea and tachypnea. These changes collectively cause “restrictive” ventilatory dysfunction, explaining why respiratory distress can be an early prominent symptom of PRRSV infection.

Notably, pulmonary surfactant proteins are not merely passive victims but active participants in antiviral defense. This concept is well-established in other viral pneumonias; for instance, influenza virus and SARS-CoV-2 infections have been shown to impair AECII function and surfactant homeostasis, leading to similar mechanical complications (24, 25). Studies show SP-A and SP-D can bind PRRSV and inhibit PRRSV infection of Marc-145 cells (26). Beyond direct neutralization, SP-A and SP-D act as pattern recognition molecules that modulate alveolar macrophage function and inflammation, further contributing to host defense against respiratory viruses (27, 28).

This finding indicates surfactant proteins are vital components of the pulmonary innate immune system, possessing direct antiviral activity against PRRSV. Particularly, recombinant porcine SP-A protein significantly inhibits PRRSV infection *in vitro*, demonstrating anti-PRRSV activity. This suggests PRRSV may downregulate these antiviral surfactant proteins to establish effective pulmonary infection. This surfactant protein dysregulation—SP-D upregulation and SP-C downregulation—may reflect complex virus-host interactions: SP-D upregulation could represent a host defense response to viral infection, while SP-C downregulation might be a viral strategy to disrupt alveolar stability.

## PRRSV-induced airway mucus hypersecretion and physical obstruction

Beyond disrupting the pulmonary surfactant system, PRRSV infection further exacerbates ventilatory dysfunction by inducing airway mucus hypersecretion. Clinical observations note that PRRSV-infected pigs often exhibit increased respiratory mucus secretion, forming viscous plugs that physically obstruct small airways. This mucus hypersecretion may be mediated by multiple mechanisms. Pro-inflammatory cytokines released after PRRSV infection of alveolar macrophages may stimulate goblet cell hyperplasia and hypersecretion. In particular, cytokines such as IL-1β and TNF-α, which are elevated in PRRSV infection and known to drive mucus production in other respiratory viral infections, could play a central role (29, 30). PRRSV-induced dysregulation of tight junction proteins not only affects vascular permeability but may also influence airway epithelial barrier function and secretory properties. Viral stress responses may further amplify mucus secretion via neuroendocrine pathways.

Airway mucus hypersecretion leads to functional airway narrowing and increased airflow resistance, creating an “obstructive” ventilatory defect. This combines with the “restrictive” ventilatory defect from surfactant deficiency, forming the “mixed ventilatory mechanics failure” characteristic of PRRSV infection. Mucus plugs in small airways not only increase airflow resistance but also create a microenvironment favorable for secondary bacterial infections, synergizing with PRRSV’s known immunosuppressive effects to worsen disease progression. This mechanism also explains why PRRSV infection is frequently accompanied by severe bacterial bronchopneumonia and shows incomplete response to antibiotic therapy.

## Integrated pathophysiological vicious cycle

PRRSV’s pulmonary destruction does not result from isolated actions of single mechanisms but from a vicious cycle of mutually exacerbating processes. Surfactant deficiency-induced alveolar collapse further increases airway resistance, while airway mucus obstruction exacerbates alveolar epithelial damage through local inflammation. More critically, pulmonary edema resulting from microvascular endothelial barrier disruption further dilutes and inactivates residual surfactant, forming a vicious cycle. Recent research provides molecular explanations for this cycle: IL-1β and TNF-α (rather than viral particles themselves) secreted by PRRSV-infected alveolar macrophages are key components driving increased vascular permeability. These inflammatory factors promote nuclear entry of three transcription factors—ILF2, GTF3C2, and THRAP3—regulating claudin-8 and claudin-4 transcription; simultaneously, they upregulate microRNA ssc-miR-185 targeting claudin-8 to inhibit its post-transcriptional expression. This mechanism directly links inflammatory responses to pulmonary barrier function disruption (11). Conversely, abnormal surfactant protein expression participates in this cycle. Studies find that SP-D overexpression inhibits SP-C promoter activity, thereby suppressing SP-C expression. As a crucial hydrophobic surfactant protein, SP-C downregulation further impairs alveolar stability, worsening alveolar collapse (21).

## Paradigm shift in therapeutic strategy: from anti-inflammation to lung protection

Current strategies for managing PRRSV respiratory symptoms primarily focus on anti-inflammatory approaches and antibiotic use. These methods target secondary disease changes rather than core pathophysiological mechanisms, often yielding limited efficacy. Research shows that even with antibiotics like tilmicosin, florfenicol, or doxycycline, treatment of PRRSV-infected pigs often fails because antibiotics cannot repair damaged lung infrastructure (31–33).

We propose shifting toward a core strategy centered on “lung protection and functional restoration”. While conceptually promising, surfactant replacement therapy requires rigorous preclinical validation in porcine models. Future studies should assess the pulmonary distribution, metabolic kinetics (pharmacokinetics/pharmacodynamics), and efficacy of exogenous surfactants (natural vs. synthetic) delivered via nebulization (optimizing particle size and frequency) in improving lung compliance and gas exchange in PRRSV-infected pigs. This includes surfactant replacement therapy using nebulized porcine-derived or synthetic surfactants—particularly indicated for high-value breeding animals—to directly replenish pulmonary surfactant, improve lung compliance, and potentially exert antiviral effects via exogenous surfactant proteins. Concurrently, mucolytic agents (e.g., N-acetylcysteine) and cilio-stimulatory compounds can be incorporated into feed or water to enhance airway clearance during outbreaks. Furthermore, immunometabolic interventions such as AMPK agonists may reprogram cellular metabolism to restore surfactant synthesis, while agents stabilizing pulmonary tight junctions could counter vascular leakage caused by PRRSV. Lastly, targeted nebulized delivery of recombinant cytokines or protective proteins enables localized treatment while minimizing systemic exposure.

## Conclusion and future perspectives

We propose that PRRSV infection induces respiratory distress primarily through targeted disruption of the pulmonary surfactant system and induction of airway mucus hypersecretion, leading to alveolar mechanical failure and airway obstruction. To substantiate this hypothesis, future confirmatory studies should include (1): precise quantification of AECII infection rates *in vivo* using flow cytometry or laser capture microdissection combined with qPCR (2); systematic evaluation of dynamic changes in bronchoalveolar lavage fluid (BALF) surfactant phospholipid composition and surface tension activity at different time points post-infection; and (3) direct measurement of lung mechanical properties (e.g., pressure-volume curves) in PRRSV-infected SPF pigs. This perspective expands PRRSV pathogenesis research from inflammation and immunosuppression to biophysical and mechanobiological dimensions. Although this viewpoint requires further experimental validation, it offers promising directions for developing novel therapeutic strategies against PRRSV respiratory symptoms.

Shifting therapeutic focus from “anti-inflammation” to “pro-homeostasis” represents a fundamental rethinking of PRRSV respiratory pathology and may provide insights into lung injury caused by other viral respiratory diseases. Establishing this new paradigm will rely on interdisciplinary collaboration, integrating knowledge from virology, immunology, pulmonary physiology, and biomechanics, ultimately opening new avenues for controlling PRRSV respiratory symptoms.
